# Generation and Characterization of a Dual-Reporter Transgenic *Leishmania braziliensis* Line Expressing eGFP and Luciferase

**DOI:** 10.3389/fcimb.2019.00468

**Published:** 2020-01-22

**Authors:** Rohit Sharma, Paulo S. Silveira-Mattos, Vinicius C. Ferreira, Francys A. Rangel, Laíse B. Oliveira, Fabiana S. Celes, Sayonara M. Viana, Mary E. Wilson, Camila I. de Oliveira

**Affiliations:** ^1^Instituto Gonçalo Moniz, FIOCRUZ, Salvador, Brazil; ^2^Departments of Microbiology and Immunology and Internal Medicine, University of Iowa, and the Veterans' Affairs Medical Center, Iowa City, IA, United States; ^3^INCT—Instituto de Investigação em Imunologia, São Paulo, Brazil

**Keywords:** *L. braziliensis*, transgenic, dual reporters, luciferase, eGFP

## Abstract

In this study, we generated a transgenic strain of *Leishmania braziliensis*, an etiological agent associated with a diversity of clinical manifestations of leishmaniasis ranging from localized cutaneous to mucocutaneous to disseminated disease. Transgenic parasites expressing reporter proteins are valuable tools for studies of parasite biology, host-pathogen interactions, and anti-parasitic drug development. To this end, we constructed an *L. braziliensis* line stably expressing the reporters eGFP and luciferase (eGFP-LUC *L. braziliensis)*. The integration cassette co-expressing the two reporters was targeted to the ribosomal locus (*SSU*) of the parasite genome. Transgenic parasites were characterized for their infectivity and stability both *in vitro* and *in vivo*. Parasite maintenance in axenic long-term culture in the absence of selective drugs did not alter expression of the two reporters or infection of BALB/c mice, indicating stability of the integrated cassette. Infectivity of eGFP-LUC, *L. braziliensis*, both *in vivo* and *in vitro* was similar to that obtained with the parental wild type strain. The possibility of *L. braziliensis* tracking and quantification using fluorescence and luminescence broadens the scope of research involving this neglected species, despite its importance in terms of public health concerning the leishmaniasis burden.

## Introduction

Leishmaniasis is a neglected tropical disease caused by the protozoan pathogen *Leishmania spp*. that is transmitted by sand flies. Nearly 1.3 million new cases occur each year (Burza et al., 2018) and 350 million people face risk of infection. The two most common forms of leishmaniasis are visceral leishmaniasis (VL) and cutaneous leishmaniasis (CL). In Brazil, *Leishmania braziliensis* is the leading cause of CL, which mostly manifests as localized lesions on the skin, but may also metastasize to mucosal sites (Bittencourt and Netto, 1995). Another common manifestation is disseminated leishmaniasis (Carvalho et al., 1994), which is characterized by the presence of large numbers of papular and acneiform ulcers affecting different parts of the body. In contrast to American Cutaneous Leishmaniasis, disseminated leishmaniasis is associated with impaired cellular immune responses, as documented by decreased CD4+ T cells and absent T cell responses to leishmania antigen (Carvalho et al., 1994). The latter form of leishmaniasis is growing in frequency in Brazil (Jirmanus et al., 2012).

The control of *Leishmania* infection relies on chemotherapy with pentavalent antimonials, miltefosine or amphotericin B, whose use is limited by significant toxicity and, in some cases, resistance (Vanaerschot et al., 2014; Ponte-Sucre et al., 2017). Since no vaccine is currently available, the identification of novel drug targets remains a priority in leishmaniasis research. To this end, reporter gene technology facilitates the high-content screening of new compounds, thereby accelerating drug discovery (Dube et al., 2009). To date, different reporter genes, such as green fluorescent protein (GFP) (Chan et al., 2003), eGFP (Mehta et al., 2008), firefly luciferase (Lang et al., 2005), and DsRed (Paape et al., 2008), have been introduced into *Leishmania spp*. via transgenesis. The resulting transgenic parasites have been successfully employed in drug screening, vaccine development and also in studies aimed at furthering the understanding of host-parasite interactions (Peters et al., 2008; Millington et al., 2010). Regarding the latter, the combined use of fluorescent transgenic parasites and fluorescent transgenic reporter mice can particularly contribute the understanding of the initial interactions between leishmania parasites and the various components of the skin microenviroment, for example Peters et al. (2008).

We have previously reported the development of a *L. braziliensis* transgenic line expressing both mCherry and luciferase (Novais et al., 2013). However, fluorescence expression in amastigotes was found to be lower than in promastigotes, limiting downstream applications of this parasite line. Therefore, we developed a transgenic *L. braziliensis* reporter line that stably co-expresses eGFP and luciferase (Luc), which was evaluated with respect to infectivity, both *in vitro* and *in vivo*. Here we show that this dual-reporter *L. braziliensis* transgenic line demonstrates promising potential for use in the study of host-parasite interactions as well as high-throughput drug screening assays. Thus, this parasite line could be very useful, especially considering that *L. braziliensis* is an important etiological agent of all tegumentary forms of leishmaniasis in Brazil.

## Materials and Methods

### Ethics Statements

Female BALB/c mice, 6–8 weeks of age, were obtained from IGM/FIOCRUZ animal facility where they were maintained under pathogen-free conditions. All animal work was conducted according to the Guidelines for Animal Experimentation of the Colégio Brasileiro de Experimentação Animal and of the Conselho Nacional de Controle de Experimentação Animal. The local Ethics Committee on Animal Care and Utilization (CEUA) approved all procedures involving animals (CEUA-012/2016-IGM/FIOCRUZ).

### Parasite Culture

*L. braziliensis* promastigotes (MHOM/BR/01/BA788) (de Moura et al., 2005) were maintained in Medium 199 (Sigma-Aldrich) supplemented with 20% heat-inactivated fetal calf serum (FCS), Hepes (40 mM), Adenine (0.1 mM), Hemin (5 μg/ml), Biotin (1 μg/ml), and antibiotics (penicillin 100 IU/mL and streptomycin 100 μg/mL) (all from ThermoScientific) at 26°C. Prior to *in vitro* and *in vivo* infection assays, selected *L. braziliensis* transfectants were grown in Schneider‘s insect medium (Sigma-Aldrich) supplemented with 10 % FCS, 2 mM L-Glutamine, Penicillin 100 U/ml and Streptomycin 100 μl/ml (ThermoScientific).

#### Generation of a Transgenic Line of *L. braziliensis* Co-expressing eGFP and Luciferase

The genes encoding firefly luciferase or a leishmania sequence-optimized gene encoding eGFP were cloned into pIR1SAT, a genomic integrating vector that was kindly provided by Stephen M. Beverley of Washington University, St. Louis, MO, USA. The leishmania-optimized eGFP gene was amplified by PCR from pIR1SAT-eGFP, also kindly provided by S. M. Beverley, using primers to generate restriction enzyme-compatible sites. The firefly luciferase gene was cloned into the *XbaI* site, and the eGFP gene was cloned into the *BglII* site and used for bacterial transformation. After selecting single colonies on LB-Ampicillin plates, cloning was verified by restriction enzyme digestion, and sequencing across cloning sites. pIRSAT-LUC_eGFP was linearized with Swa*I* and purified using gel extraction kit (GE healthcare Life Sciences). For the transfection, wild type *L. braziliensis* promastigotes (log phase) were centrifuged, resuspended in Tb-BSF buffer (Schumann Burkard et al., 2011) at a density of 10^8^ cells/ml. Ten microgram of purified linear plasmid (50 μl) were mixed with 350 μl (ca. 4 × 10^7^ cells) in a pre-chilled 2-mm cuvette (BIO-RAD). Electroporation was performed as previously described (Kapler et al., 1990) using Genepulser (BIO-RAD). Electroporated cells were incubated in ice for exactly 10 min, transferred to 10 ml M199 medium and incubated for 24 h at 26°C. The transfected cells were then gently dispersed onto solid M199 plates (2% noble agar) supplemented with Nourseothricin (50 μg/ml) (Thermo) and incubated for 10–12 days at 26°C. Nine randomly picked colonies were cultured in 1 ml of M199 (Thermo) medium supplemented with Nourseothricin (50 μg/ml) for 2–3 days, and microscopically examined for growth and flagellar motility Genomic DNA was extracted (Qiagen) from two resulting clones and selected based on fluorescence intensity, using commercially available kits. An integration diagnostic PCR was performed using primers IntF (5′-ACG ATT AGA GAC ACA AAC GAA CAA-3′) and IntR (5′-ATA TAG AAT AAG CCT CCC CGA GTT-3′), specific for the nourseothricin gene resistance gene (SAT), present within the integration cassette and the ribosomal locus of *L. braziliensis* genome, respectively. PCR cycle conditions were as follows: 94°C for 2 min, 94°C for 30 s, 50°C for 30 s, 72°C for 90 s (for 35 cycles), 72°C for 5 min (1 cycle). The PCR product was separated on a 1% agarose gel, purified and cloned into a pGEM-T Easy vector, according to manufacturer's instructions (Promega). Positive clones were sequenced using vector specific T7 and SP6 primers and were examined for eGFP expression by fluorescence microscopy.

### Luciferase Assay and eGFP Detection

Mid-log phase eGFP-LUC *Lb* promastigotes were washed twice with PBS assayed for Luciferase activity using ONE-Glo™ Luciferase Assay (Promega), according to manufacturer's instructions and as described elsewhere (Reimao et al., 2013). Luminescence was measured in a microplate reader (Molecular Devices). Experiments were performed in triplicate. eGFP expression was evaluated by flow cytometry, data (10,000 events) were acquired on a Fortessa flow cytometer (BD Biosciences) and analyzed using FlowJo (Tree Star Inc.). Wild type (WT) *L. braziliensis* was used as the negative control.

### Animal Infection

BALB/c mice (*n* = 6) were inoculated with 10^5^ *L. braziliensis* expressing GFP and Luciferase (eGFP-LUC *Lb*) promastigotes, enriched for the presence of metacyclics as described elsewhere (da Silva et al., 2015). Parasites were inoculated into the left ear dermis in 10 μl PBS using a 27G needle. Lesion development was monitored weekly by measuring the thickness of the ear using a digital caliper (Thermo). Parasite loads were determined by limiting dilution analysis from samples obtained from the ear and draining lymph nodes, at weeks 6 and 10 post parasite inoculation, as described (Novais et al., 2013). Parasites recovered following *in vivo* passages were used in subsequent *in vitro* experiments.

### Infection of Bone-Marrow Derived Macrophages With eGFP-LUC Lb

Macrophages (BMDM) were differentiated from mouse bone marrow as described (Weischenfeldt and Porse, 2008). Cells were resuspended in RPMI 1640 medium (SIGMA) supplemented with 100 U/ml penicillin, 100 μg/ml streptomycin and 10% FBS for seeding onto glass coverslips, each at 3 × 10^5^ cells/500 μl/coverslip in 24-well plates. After cell attachment, monolayers formed on the coverslips were washed to remove non-adherent cells and were cultured with 3 × 10^6^ eGFP-LUC *Lb* promastigotes (10:1 parasite/host cell) in supplemented RPMI at 37°C, 5% CO_2_. After 6 and 24 h, coverslips with monolayers were extensively washed to remove non-internalized parasites, fixed and stained with hematoxylin and eosin. Parasite uptake was determined by microscopic counting of 200 macrophages, in quintuplicate, for the number of cells with and without *Leishmania*, and the total number of intracellular *Leishmania*. Fluorescent parasites observed in an inverted Leica DMi8 platform (Leica Microsystems).

### Treatment of eGFP-LUC Lb-Infected BMDM With Amphotericin B

BMDM were infected with eGFP-LUC *Lb* promastigotes as described above. Infected cells were then treated with Amphotericin B (Sigma) (0.25 μg/ml) for 24 h. In some experiments, cells were cultured in polypropylene tubes and assayed for the percentage of eGFP-expressing macrophages by flow cytometry. Cells were resuspended in FACS buffer (PBS supplemented with 1% fetal bovine serum) and data (10,000 events) were acquired on a Fortessa flow cytometer (BD Biosciences) and analyzed using FlowJo (Tree Star Inc.). Alternatively, cells were resuspended in PBS and assayed for luciferase activity as described above. For each experiment, background luminescence was calculated using WT *L. braziliensis*-infected macrophages and control uninfected macrophages. Assays were performed in triplicate coverslips per experiment.

### Statistical Analysis

Comparisons between two groups were performed by Mann-Whitney (non-parametric *t*-test). Analyses were conducted using Prism (GraphPad, V, 8.0) and a *p* ≤ 0.05 was considered significant.

## Results

### Generation of *L. braziliensis* Mutant Expressing eGFP and Luciferase

**Figure 1:**
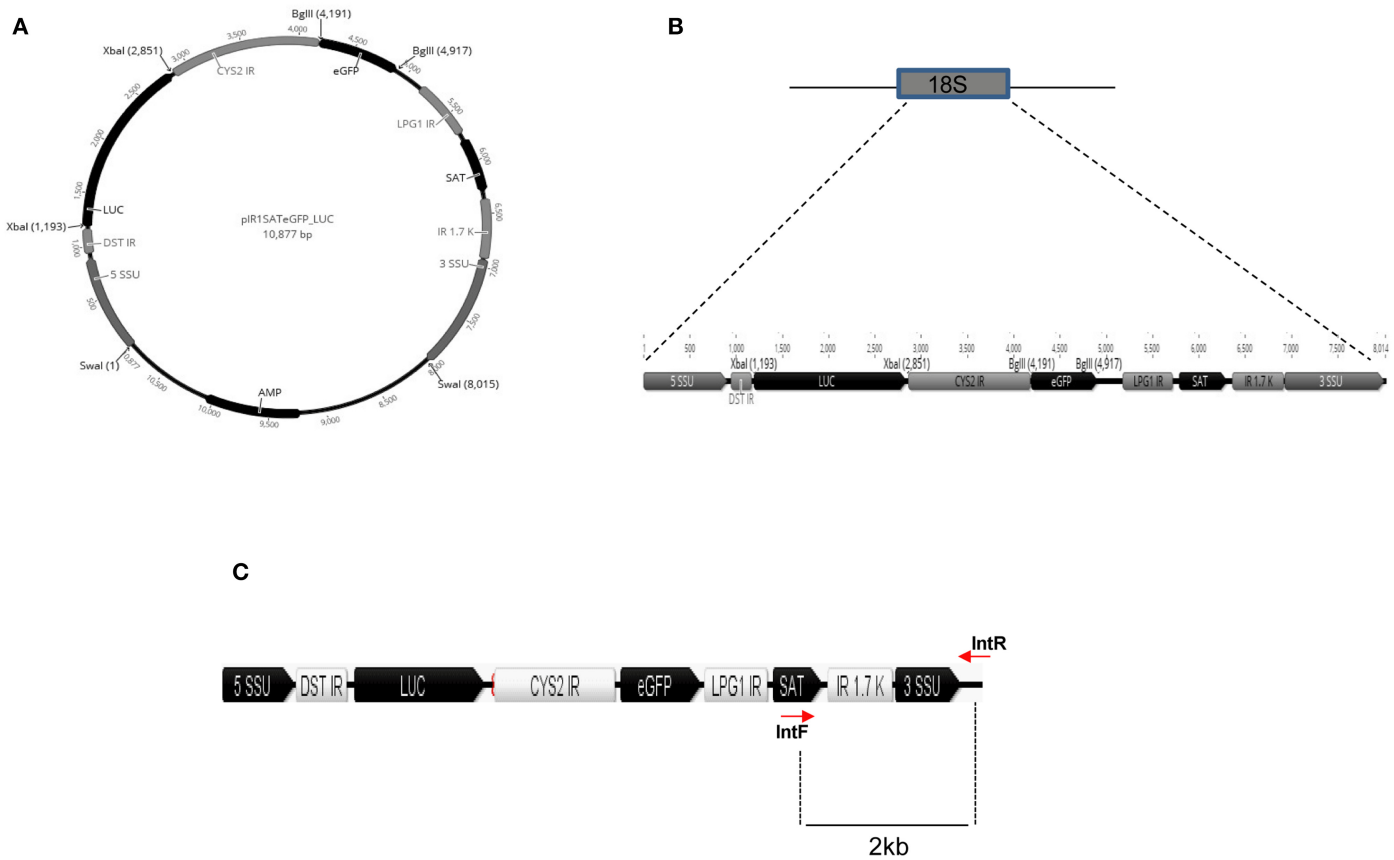
In silico construct of pIRSATeGFP_LUC. **(A)** Luciferase and eGFP genes cloned between the *XbaI* and *BglII* restriction sites, respectively. **(B)** Schematic representation of the SSU integration locus and the cassette for integration with eGFP and LUC ORF. **(C)** Schematic representation of the linear cassette for integration in the SSU rDNA locus. SSU, small subunit, DST IR, intergenic region of the DST gene, LUC, Luciferase, CYS2 IR, intergenic region of cysteine protease 2 gene, eGFP, enhanced GFP, LPG1R, intergenic region of the LPG1 gene, SAT, nourseothricine resistance gene, IR 1.7K, intergenic region of dihydrofolate reductase-thymidylate symthase locus. Position of the primers and size of amplified fragments are indicated by lines.

To generate a *L. braziliensis* line co-expressing eGFP and Luciferase (eGFP-LUC *Lb*), we employed a plasmid containing the eGFP and LUC genes, cloned into sites of pIR1-Sat that are flanked by 5′ and 3′SSU sequences of *Leishmania spp*. (Figures 1A,B). As such, integration of the eGFP and Luciferase cassettes into the SSU locus would be confirmed by generating the expected 2.0 Kb band (Figure 1C).

**Figure 2:**
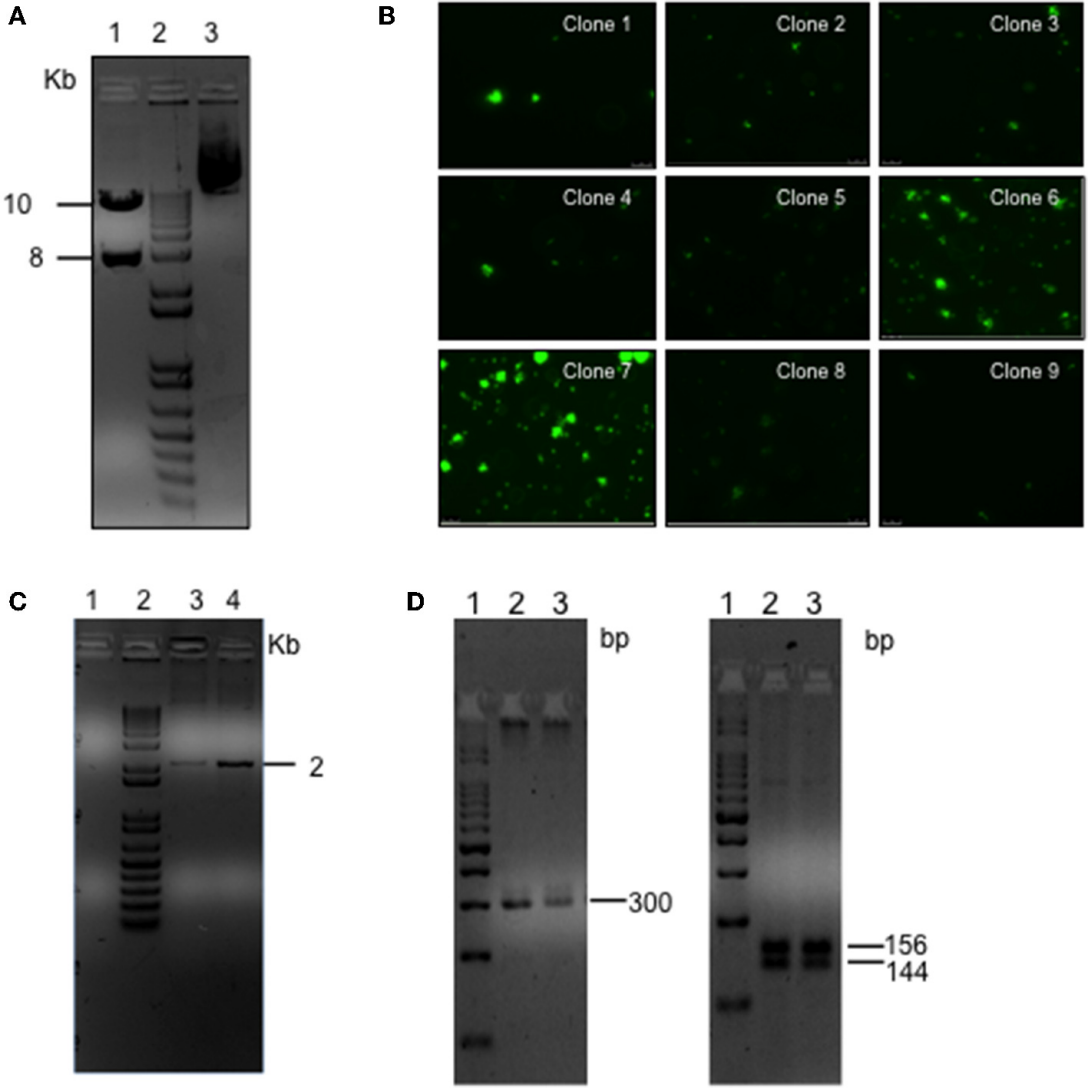
Generation of the *L. braziliensis* co-expressing eGFP and LUC. **(A)** Agarose gel electrophoresis of pIRSATeGFP_LUC following Swa*I* digestion (lane 1), 1Kb marker (lane 2), Undigested circular plasmid (lane 3). **(B)** Fluorescent microscopy of transgenic *L. braziliensis clones*. **(C)** Agarose gel electrophoresis of PCR product amplified using primers complementary to the Nourseothricin resistance marker (SAT) present in the of integration cassette and complimentary to the rDNA locus of the *L. braziliensis* genome (outside the cassette), indicative of eGFP-LUC integration, SSU locus (18s gene). PCR was performed using genomic DNA purified from WT *Lb* (lane 1) or from transgenic *Lb* (clones 6 and 7) (lane 3 and 4, respectively). **(D)** Agarose gel electrophoresis of PCR product amplified using primers complimentary to the ITS-1 locus using genomic DNA purified from WT-*Lb* (lane 2) or eGFP-LUC *Lb* (transfected clone 7) (lane 3) (Left panel). Hae*III* digestion of ITS-1 PCR product. WT-Lb (lane 2) or transgenic Lb (transfected clone 7) (lane 3) (right panel).

*L. braziliensis* promastigotes were transfected with the linearized plasmid containing the eGFP and LUC genes, cloned into sites of pIR1-Sat that are flanked by 5′ and 3′SSU sequences of *Leishmania spp*. (Figure 2A). After transfection, parasites were grown in an antibiotic-selective medium and nine independent clones were randomly selected and evaluated for eGFP expression by fluorescence microscopy. Two of these clones (clones #6 and #7) displayed a strong signal (Figure 2B). Proper integration of the eGFP and Luciferase cassettes into the SSU locus was confirmed by PCR of parasite genomic DNA, which yielded the expected 2.0 Kb band (Figure 2C). As an additional confirmation, the purified PCR amplicon was cloned into a p-GEMT vector and sequenced using vector-specific primers (Supplementary Figure 1). Clone #7 was chosen based on the strongest GFP signal. Genotypes of isolated colonies were verified using ITS-1 amplification followed by RFLP (Schonian et al., 2003) and yielded the expected 300 bp fragment and the 144 and 156 bp segments following restriction enzyme digestion (Figure 2D), as did wild-type (WT) *L. braziliensis*. Sequencing of the ITS-1 amplicon demonstrated high identity (97%) of eGFP-LUC and WT *L. braziliensis* (*Lb)* (Genebank Id- FN398335.1; MHOM/BR/2002/NMT-RBO018) (Supplementary Figure 2). These results demonstrate the successful integration of this dual-reporter system containing eGFP and Luc into the *Lb* genome.

### *In vitro* Detection of eGFP and Luciferase in eGFP-LUC *Lb* Promastigotes

**Figure 3:**
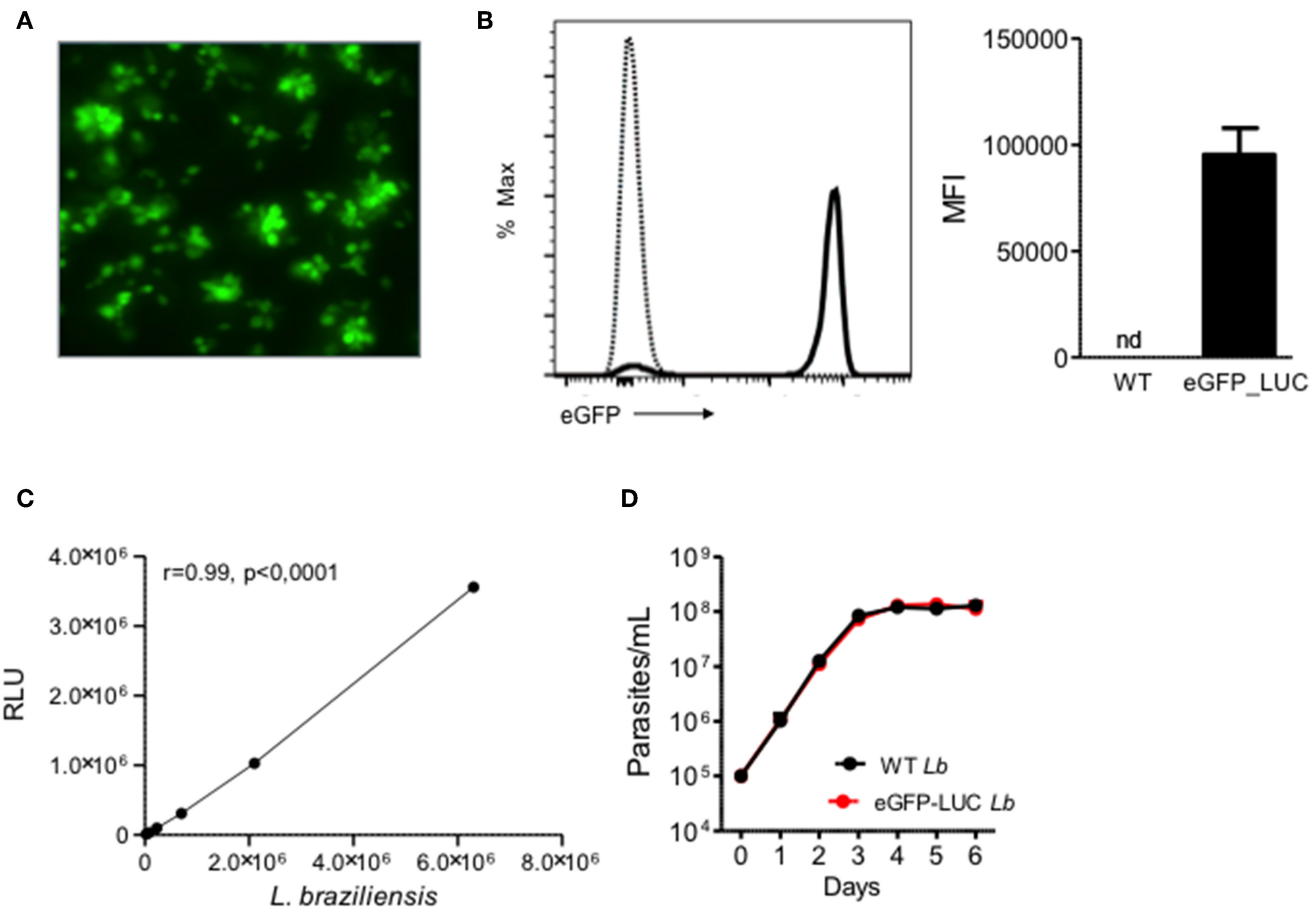
Expression of eGFP and Luciferase in transgenic *L. braziliensis*. **(A)** Fluorescence microscopy of transgenic *L. braziliensis* (clone #7) promastigotes. **(B)** Representative histogram depicting eGFP in WT *L. braziliensis* (dotted line) and eGFP-LUC *L. braziliensis* (clone #7) (solid line), both shown as % of Max. Bar graph represents the MFI of WT and eGFP-LUC *L. braziliensis*. Data (mean ± SEM) are from a representative experiment. **(C)** Luminescence intensity and number of eGFP-LUC *L. braziliensis* promastigotes. Promastigotes were serially diluted and luminescence was measured using a microplate reader, after addition of luciferin. RLU, relative light units. **(D)** Growth curve of WT and eGFP-LUC *L. braziliensis* (clone #7). Parasites were cultures in supplemented Schneider medium and parasites numbers were determined daily using a hemocytometer. Data (mean ± SEM) are from a representative experiment, performed in quintuplicate. nd, not detected.

As expected, eGFP-LUC *Lb* recovered from infected mice displayed GFP fluorescence by both microscopy (Figure 3A) and flow cytometry (Figure 3B). In parallel, luminescence of eGFP-LUC *Lb* was also confirmed by incubation with luciferin and a linear correlation between the number of promastigotes and light production (Figure 3C) was observed. Lastly, the *in vitro* growth rates of WT *Lb* and eGFP-LUC *Lb* promastigotes recovered from infected mice were similar (Figure 3D) indicating that the transgenic eGFP-LUC *Lb* line did not exhibit compromised parasite growth after *in vivo* experimentation.

### Course of Infection of *eGFP-LUC Lb* in BALB/c Mice

**Figure 4:**
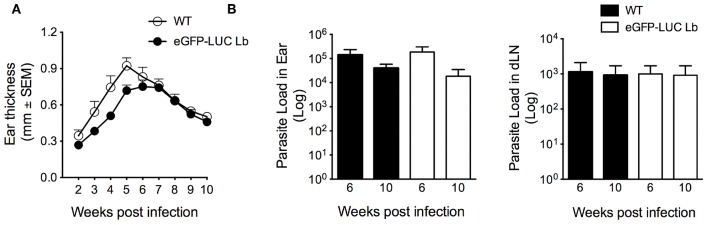
*In vivo* infectivity of transgenic *L. braziliensis* co-expressing eGFP and Luciferase. **(A)** BALB/c mice **(***n* = 6) were infected with enriched metacyclics of WT or eGFP_LUC Lb (10^5^) and lesion development was measured weekly. **(B)** Parasite load was determined at the infection site and at the dLN by limiting dilution analysis. Data (mean ± SEM) are from a representative experiment, performed with 6 mice.

Following the demonstration of GFP expression by fluorescence microscopy and confirmation of integration of the dual reporter system (Figure 1), eGFP-LUC *Lb* promastigotes (clone #7) were serially passaged *in vitro* in the absence of selective drug pressure. We then evaluated the *in vivo* development of cutaneous lesions. Accordingly, BALB/c mice were inoculated in the ear dermis with eGFP-LUC *Lb* enriched metacyclics. Similarly to WT *Lb*, mice infected with eGFP-LUC *Lb* developed lesions that peaked at 5-6 weeks post-parasite inoculation (Figure 4A). Moreover, parasite load, determined by limiting dilution analysis, was similar comparing WT *Lb* vs. eGFP-LUC *Lb*, both at the inoculation site and at draining lymph nodes, at two different time points (Figure 4B). These data indicate that *in vivo* parasite growth in this transgenic cell line is similar to that reported for WT *Lb*. The eGFP-LUC *Lb* recovered from the draining lymph nodes of infected mice was further evaluated *in vitro*.

### Infection of BMDM With eGFP-LUC *Lb*

**Figure 5:**
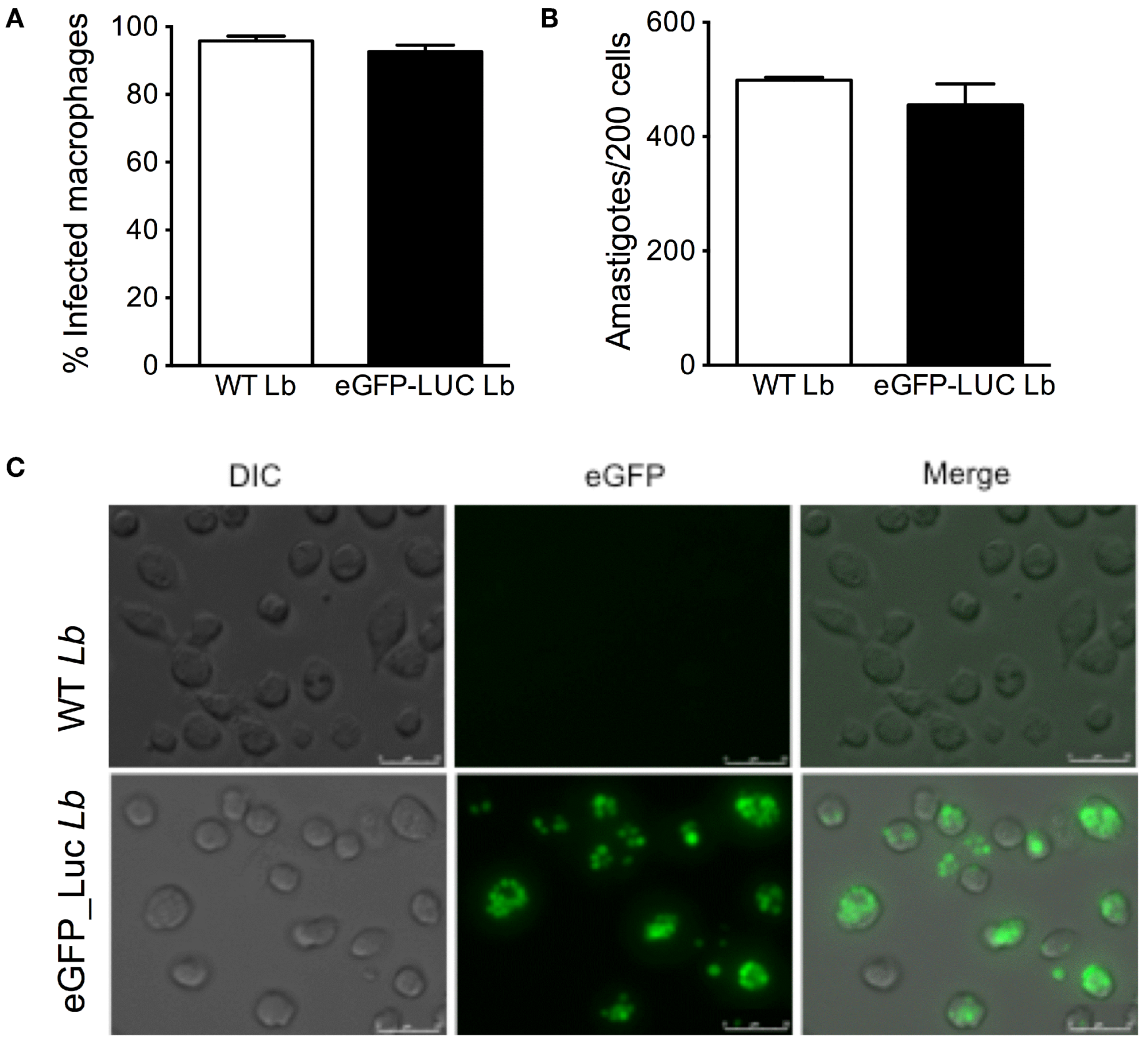
*In vitro* infectivity of transgenic *L. braziliensis* co-expressing eGFP and Luciferase. Bone-marrow derived macrophages were infected with either WT or eGFP-LUC *L. braziliensis* (clone #7), for 4 h. Cells were processed for microscopy to assess the **(A)** percentage of infected cells and **(B)** the number of amastigotes per 200 macrophages Data (mean ± SEM) are from a representative experiment, performed in quintuplicate. **(C)** DIC, fluorescent (eGFP) and merged micrographs of macrophages infected with WT or eGFP-LUC *L. braziliensis* for 24 h.

We then assayed the infection rate of eGFP-LUC *Lb* in BMDM by optical microscopy and we observed similar percentages of infected cells and numbers of amastigotes, comparing the parental line and eGFP-LUC *Lb* (Figures 5A,B). Fluorescence microscopy confirmed the feasibility of employing eGFP-LUC *Lb* for the quantification of infected macrophages also suggested the possibility of using this cell line for determination of parasite load (Figure 5C). Therefore, *in vitro* and *in vivo* passages did not compromise the infectivity of eGFP-LUC *Lb*, compared to the expected outcomes reported for WT *L. braziliensis*.

**Figure 6:**
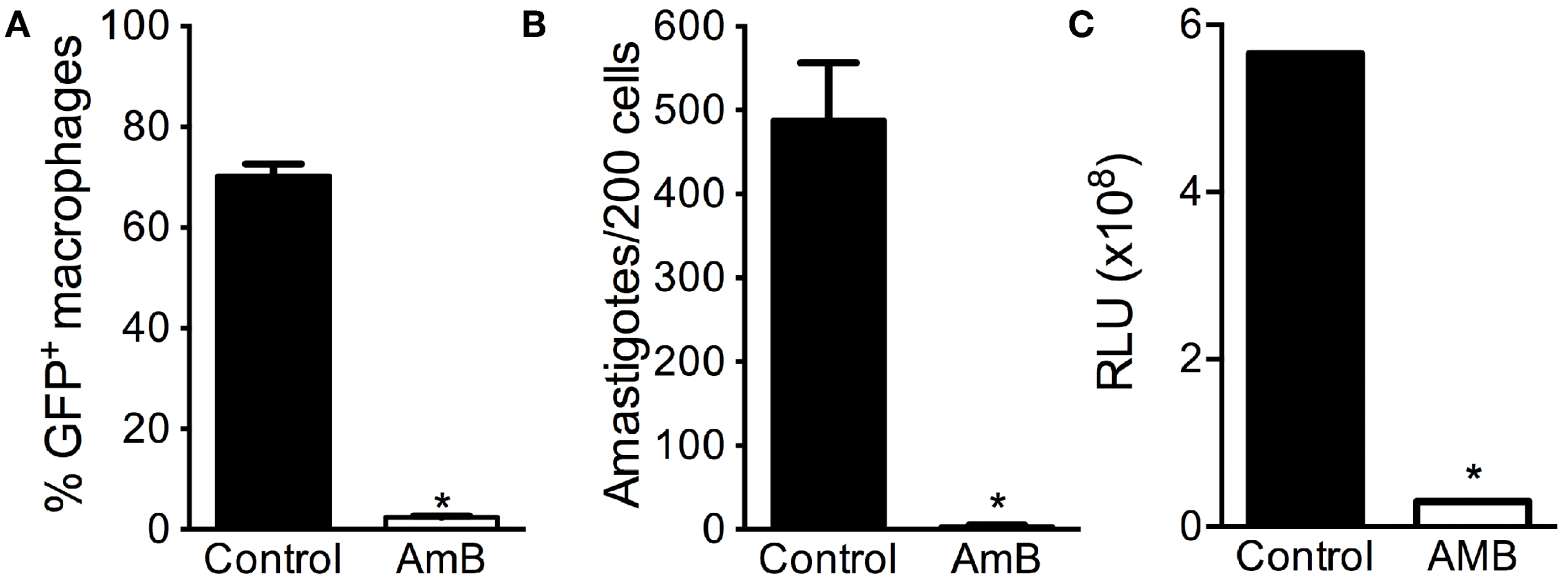
Applicability of eGFP-LUC *L. braziliensis* for drug screening. Bone-marrow derived macrophages were infected with either WT or eGFP-LUC *L. braziliensis* (clone #7), for 24 h. Cells were treated with Amphotericin B and assayed by flow cytomery to determine **(A)** the percentage of infected cells. Infected cells were also stained with H&E and the number of amastigotes per 200 macrophages was determined by optical microscopy **(B)**. Luminescence intensity of eGFP-LUC *Lb*-infected macrophages was measured using a microplate reader, after addition of luciferin **(C)**. RLU, relative light units. Data (mean ± SEM) are from three experiments experiment, each performed in triplicate. * indicates statistically significant difference.

Lastly, we probed for the feasibility of using eGFP-LUC *Lb* in drug screening assays. BMDM were infected with eGFP-LUC *Lb* and exposed to Amphotericin B. As expected, the percentage of eGFP-LUC *Lb*-infected macrophages (Figure 6A) and the number of intracellular amastigotes (Figure 6B) decreased significantly, as determined by optical microscopy and by flow cytometry, respectively. We also observed a significant decrease in luminescence levels following treatment of eGFP-LUC *Lb-*infected BMDM with Amphotericin B (Figure 6C). Collectively, these findings demonstrate that our dual-reporter eGFP-LUC *Lb* cell line can be employed in assays aimed at drug screening, for example, involving either fluorescence or luminescence.

## Discussion

For a number of years now, the use of transgenic protozoan parasites expressing reporter genes has proven valuable in understanding the biology of *Leishmania* spp. parasites as well as host-parasite interactions (Amino et al., 2007; Beattie et al., 2008; Di Cristina et al., 2008). In spite of the status of *L. braziliensis* as a neglected species, and considering that this species is largely responsible for most cases of CL, we sought to generate a transgenic dual-reporter line co-expressing eGFP and luciferase from a clinical isolate. The resulting transgenic line was then tested both *in vitro* and *in vivo* in a mouse model of CL.

We initially confirmed the stable integration of the eGFP-luciferase expression cassette in *L. braziliensis* transfectants. Although, this species has maintained its functional RNAi machinery (Lye et al., 2010), which may present a challenge to episomal expression of constructs, an integrated dual reporter cassette should provide a more robust an reliable tool for future studies. This successful integration of the reporter cassette within the constitutively expressed SSU locus allowed us to co-express both eGFP and luciferase reporters. Expression of both transgenes was stable after >10 serial passages *in vitro* and levels of both eGFP and luciferase also remained unaltered after parasites were recovered infected BALB/c mice. Importantly, ear thickness and parasite load in BALB/c mice inoculated with eGFP-LUC *Lb* were similar compared to those obtained after infection with the parental WT *L. braziliensis* (de Moura et al., 2005). These results indicate that our transgenic dual-reporter *L. braziliensis* line exhibits similar behavior as its parental strain *in vivo*, and did not lose virulence during *in vitro* manipulation.

We then confirmed that eGFP-LUC *Lb* parasite retained its infectiveness in BMDM: the percentage of infection and the number of intracellular amastigotes was similar compared to parental WT *L. braziliensis*. Numbers of amastigotes were similar when assessed by light or by fluorescence microscopy, indicating we were not detecting a subset of parasites expressing either reported. Treatment of eGFP-LUC *Lb*-infected BMDM with Amphotericin B significantly reduced infection, as detected by flow cytometry and by luminescence. These findings show that *Lb* infection can be effectively monitored using both of these reporters and opens the possibility of using this line for downstream studies aimed at drug screening. Although the presence of two reporters for drug screening *in vitro* is not mandatory, the presence of a bright fluorescence marker such as eGFP allows for more robust experiments involving parasite killing in monocytic cell lines or in primary macrophages using a multi-well-based fluorescence assay and, eventually, flow cytometry to probe for intracellular localization. As a next step, one can envisage *in vivo* imaging coupled to tissue localization as a read-out for parasite presence, for example. This would be particularly interesting for *L. braziliensis* which causes mucosal (Bacellar et al., 2002) and disseminated disease (Machado et al., 2019). To this date, it remains unknown how parasite disseminate from the primary lesion to secondary sites.

Luciferase does permit bioimaging with little background interference, its use requires luciferin substrate, thereby limiting applications (Dube et al., 2009). Bioluminescence, however, allows for the quantification of parasite burden in live animals, reducing and refining their use (Kirk, 2018). The possibility of imaging parasite presence is certainly a major advantage in the study of chronic parasitic diseases. Costa et al. developed a dual-reporter transgenic *Trypanosoma cruzi* line expressing chimeric luciferase fused to mNeonGreen (Costa et al., 2018). This particular line also incorporated CRISPR/Cas9 functionality, facilitating parallel genome editing approaches. The ability to visualize interactions and track infection using transgenic parasite lines enables researchers to address key questions in parasite biology, such as differentiation and transmission. Bastos et al. reported on the construction of a *L. braziliensis* line expressing eGFP (Bastos et al., 2017), using a WHO reference strain (MHOM/BR/75/M2904) and pLEXSY for generation of the transgenic. Similar to our results, authors demonstrated that the line constitutively expresses eGFP after serial passages *in vitro* and remained susceptible to Amphotericin B treatment following macrophage infection. Coelho et al. developed a transgenic *L. braziliensis* line expressing luciferase which was validated in mice as a tool to monitor treatment with Miltefosine (Coelho et al., 2016). This was also developed based on a field isolate (MHOM/BR/94/H3227); however, authors observed a low sensitivity, making the *in vivo* detection of low parasite burdens difficult. We build on these currently available tools by developing a dual reporter-expressing *L. braziliensis*. Besides the advantage of having two stable reporters, this line was developed upon a field isolate (de Moura et al., 2005) that has been widely employed in a variety of studies addressing the pathogenesis of CL caused by *L. braziliensis* (Novais et al., 2013, 2017), in addition to drug (Santos et al., 2014; Celes et al., 2016) and vaccine development (Salay et al., 2007; Thalhofer et al., 2011; Carneiro et al., 2012).

The possibility of *in vivo* imaging using eGFP-LUC *Lb* line shall contribute to the study of the pathogenesis of CL caused by *L. braziliensis*, as this dual-reporter system enables parasite imaging *in vivo*, in different tissues and host cells, as demonstrated by other authors in *L. infantum* (Thalhofer et al., 2011; Reimao et al., 2015; Alvarez-Velilla et al., 2019) and *L. amazonensis* (Reimao et al., 2013). Besides studies focused on screening for novel drugs, those aimed at elucidating the dynamics of mucosal tropism, a hallmark of *L. braziliensis*, or parasite persistence in selected sites (Mandell and Beverley, 2017) should also benefit from this tool.

## Data Availability Statement

All datasets generated for this study are included in the article/Supplementary Material.

## Ethics Statement

The animal study was reviewed and approved by CEUA IGM-FIOCRUZ.

## Author Contributions

RS, PS-M, VF, FR, LO, FC, and SV performed experiments and analyzed data. RS, MW, and CO drafted the manuscript. MW and CO contributed reagents. RS and CO designed experiments.

### Conflict of Interest

The authors declare that the research was conducted in the absence of any commercial or financial relationships that could be construed as a potential conflict of interest.
