# Supplementary material for: Generation and Characterization of a Dual-Reporter Transgenic Leishmania braziliensis Line Expressing eGFP and Luciferase
Source: Front Cell Infect Microbiol. 2020 Jan 22;9:468. doi: 10.3389/fcimb.2019.00468 (PMC6987073; doi:10.3389/fcimb.2019.00468)
Supplement: Supplementary file 1 [file Data_Sheet_1.docx]

**Generation and characterization of a dual-reporter transgenic *Leishmania braziliensis* line expressing eGFP and luciferase**

Rohit Sharma^1^, Paulo Sergio Mattos^1^, Vinicius C. Ferreira^1^, Francys A. Rangel^1^, Sayonara M. Viana^1^, Fabiana S. Celes^1^, Laíse B. Oliveira ^1^, Mary E. Wilson ^2^, Camila I. de Oliveira^1,4^*

Supplementary Material


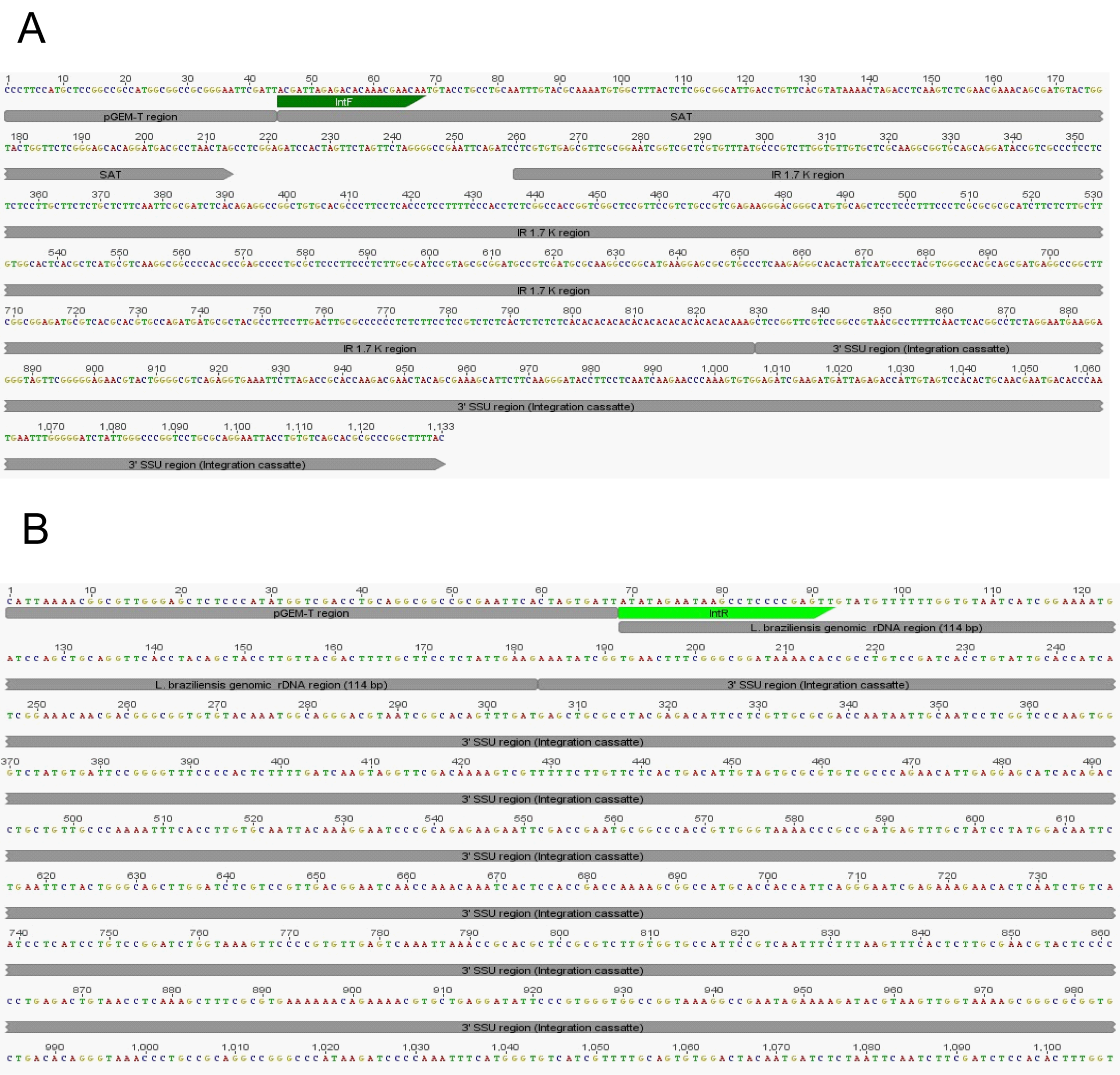


**Supplementary Figure 1**: Sequence annotation of eGFP-LUC cassete. **(A)** PCR product depicting integration of the eGFP-LUC cassete: IntF- PCR specific forward primer binding site on nourseothricin resistance gene (SAT) region, 3’ SSU region of integration cassette. (**B)** Sequence annotation of PCR product (pGEM-T vector specific SP6 primer); IntR- PCR specific reverse primer binding site on the genomic locus of *L. braziliensis* (114 bp away on the rDNA region), 3’ SSU region of integration cassette.

**Supplementary Figure 2** Sequence alignment of ITS-1 from eGFP-LUC *L. braziliensis* (clone 7) and *L. brazilensis* (Genebank Id FN398335.1; strain MHOM/BR/2002/NMT-RBO018) with calculated Identities: 0.9693878 (~ 97%).
